# Fast, large area multiphoton exoscope (FLAME) for macroscopic imaging with microscopic resolution of human skin

**DOI:** 10.1038/s41598-020-75172-9

**Published:** 2020-10-22

**Authors:** Alexander Fast, Akarsh Lal, Amanda F. Durkin, Griffin Lentsch, Ronald M. Harris, Christopher B. Zachary, Anand K. Ganesan, Mihaela Balu

**Affiliations:** 1 Beckman Laser Institute and Medical Clinic, University of California, Irvine, 1002 Health Sciences Rd., Irvine, CA 92612 USA; 2 Department of Dermatology, University of California, Irvine, 1 Medical Plaza Dr., Irvine, CA 92697 USA

**Keywords:** Biomedical engineering, Microscopy, Engineering

## Abstract

We introduce a compact, fast large area multiphoton exoscope (FLAME) system with enhanced molecular contrast for macroscopic imaging of human skin with microscopic resolution. A versatile imaging platform, FLAME combines optical and mechanical scanning mechanisms with deep learning image restoration to produce depth-resolved images that encompass sub-mm^2^ to cm^2^ scale areas of tissue within minutes and provide means for a comprehensive analysis of live or resected thick human skin tissue. The FLAME imaging platform, which expands on a design recently introduced by our group, also features time-resolved single photon counting detection to uniquely allow fast discrimination and 3D virtual staining of melanin. We demonstrate its performance and utility by fast ex vivo and in vivo imaging of human skin. With the ability to provide rapid access to depth resolved images of skin over cm^2^ area and to generate 3D distribution maps of key sub-cellular skin components such as melanocytic dendrites and melanin, FLAME is ready to be translated into a clinical imaging tool for enhancing diagnosis accuracy, guiding therapy and understanding skin biology.

## Introduction

Non-invasive clinical skin imaging with molecular contrast and high spatial resolution is important for maximizing diagnostic efficacy, guiding therapy and advancing the understanding of skin biology. The significance of technologies capable of 3D non-invasive imaging in living skin tissue is underscored by the continuous growth of in vivo Reflectance Confocal Microscopy (RCM) and more recently of the Optical Coherence Tomography (OCT) and multiphoton microscopy (MPM) in the dermatology field. RCM and OCT contrasts are based on variations in tissue refractive indices, which provide gray scale images. Gray scale images are generally sufficient for the purpose of diagnosis based on tissue morphology assessment. Therefore the RCM potential has been demonstrated for a broad range of applications in the dermatology field including non-invasive diagnosis of melanoma^1,2^ and non-melanoma skin cancer^3,4^. OCT, which generates images with lower spatial resolution than RCM and MPM, has the benefit of enhanced imaging speed, field of view and penetration depth. In the dermatology field, OCT has been proven mostly effective for non-invasive diagnosis of non-melanoma skin cancer^5,6^. The parameters of interests (spatial resolution, scanning speed, field-of-view, contrast mechanism) of the existing commercial systems for label-free clinical skin imaging based on RCM (VivaScope, Caliber ID) and OCT (VivoSight, Michelson Diagnostics) are included in Table S1, Supplementary Information, along with those for the commercial MPM clinical system (MPTflex, Jenlab) and the proposed FLAME imaging platform. MPM is a nonlinear optical imaging technique that, compared to RCM and OCT, provides unique structural and molecular contrast based on endogenous signals such as two-photon excited fluorescence (TPEF) from NAD(P)H, FAD + , melanin, keratin and elastin fibers, and second harmonic generation (SHG) from collagen^7,8^. The fluorescence lifetimes of these fluorophores, if detected, can further improve the molecular contrast^9,10^. Therefore, based on its contrast mechanism, MPM provides color images that closely resemble the histological sections dermatopathologists use for diagnosis, a significant benefit, facilitating accurate image interpretation. Furthermore, the molecular contrast of MPM images uniquely allows non-invasive, label-free evaluation of cellular metabolism in human skin^11,12^ or of skin conditions such as elastosis^13^. It also facilitates development of quantitative analyses strategies based on the TPEF and SHG signals^14–16^. However, comparing to RCM and OCT, MPM involves a more complex technology to adapt to bedside. The only MPM commercial platform optimized for clinic use (MPTflex, Jenlab, Germany) has played a critical role in establishing the clinical potential of MPM for diagnosis of skin cancer^12,14,17–20^, pigmentary skin disorders^13^ and alopecia^21^, characterization and understanding of skin pigment biology^22^ and keratinocytes metabolism^11^ or monitoring the effects of cosmetic treatments^23^. While these studies demonstrate the unique clinical potential of this technology, its routine implementation into clinical practice is hindered by several technological barriers. The main challenges are related to limited scanning area and speed. The capability of imaging large areas is critical for improving diagnostic accuracy, particularly for non-uniform pigmented lesions, such as melanoma arising in pre-existing nevus^24^. Melanocytes that may develop into melanoma in these lesions may be easily missed by imaging of limited areas. False-negative findings delay diagnosis and compromise treatment efficacy. Enhancing imaging speed is required in order to improve the efficiency of the imaging procedure, minimize motion artifacts and optimize clinical work-flow. The scientific research community has been interested in MPM rapid imaging of large tissue areas with sub-micron resolution for various applications. One area of interest includes employing MPM for rapid intraoperative assessment of thick excision specimens^25–27^. Commercial MPM microscopes can provide millimeter-to-centimeter scale sub-cellular resolution images acquired within minutes by detecting exogenous fluorescence signals, as recently demonstrated for breast^27^, prostate^25^ and skin tissue specimens^26,28^. A similar performance has been reported by several research groups in the neuroscience field, who developed customized MPM platforms for imaging mouse brain vasculature^29^, cortical tissue and neural activity^30–32^ or for in vivo brain imaging of genetically modified mouse models^33^. All the aforementioned applications involve detection of exogenous fluorescence signals. The challenge of optimizing the MPM for in vivo MPM imaging of human skin, our application of interest, is to reach a similar performance of the MPM system in terms of scanning area and speed while detecting weak endogenous signals such as those from skin fluorophores.

Successful clinical translation of the MPM technology for in vivo skin imaging applications requires optimization of the microscope complexity, cost and footprint in addition to significant improvement of scanning area, speed and contrast, without compromising spatial resolution. We have recently demonstrated a bench-top prototype of an MPM imaging system based on TPEF and SHG signals detection, optimized to image tissue areas of ~ 0.8 × 0.8 mm^2^ at speeds of less than 2 s per 1 MPx frame for high SNR, with lateral and axial resolutions of 0.5 μm and 3.3 μm, respectively^34^. In this manuscript, we present significant advances of this imaging platform, based on combining optical and mechanical scanning mechanisms with image restoration neural network computational approaches^35,36^ to allow *millimeter-to-centimeter scale* imaging within minutes, while maintaining sub-micron resolution. The main challenge of implementing a fast-scanning approach with MPM is related to the weak endogenous fluorescence signals from molecular components of skin. We have adopted a recently validated deep learning approach, namely content-aware image restoration (CARE)^35,37,38^, which represents a key solution for improving the SNR of images acquired by detecting a limited number of photons due to the high imaging speed. The proposed compact, fast large area multiphoton exoscope (FLAME) system also features time-resolved single photon counting (SPC) detection with sufficient temporal resolution to distinguish fluorophores, such as melanin, which facilitates its quantitative assessment, important in the diagnosis and the treatment evaluation of many skin conditions. A key component of this imaging platform is the light source, a femtosecond fiber laser, which has a small footprint that allows its integration into the imaging head. This allowed us to reduce the footprint of the FLAME platform 20 × compared to our previously reported bench-top prototype. We demonstrate the performance of the FLAME system by ex vivo and in vivo imaging of human skin.

## Results

### Main advances of the FLAME imaging platform

Key advances of the FLAME imaging platform with respect to the bench-top prototype previously reported by our group^34^ include: (1) significant enhancement (20 ×) of the imaging system compactness; (2) increase in the scanning speed over centimeter-scale tissue areas; (3) enhancement of the molecular contrast through specific detection of melanin. The applications enabled by these advancements, along with a brief description of the imaging platform, are presented below. The hardware and software components involved in the FLAME system development are described in the “Materials and methods” section.

#### Enhancing compactness and portability

**Figure 1:**
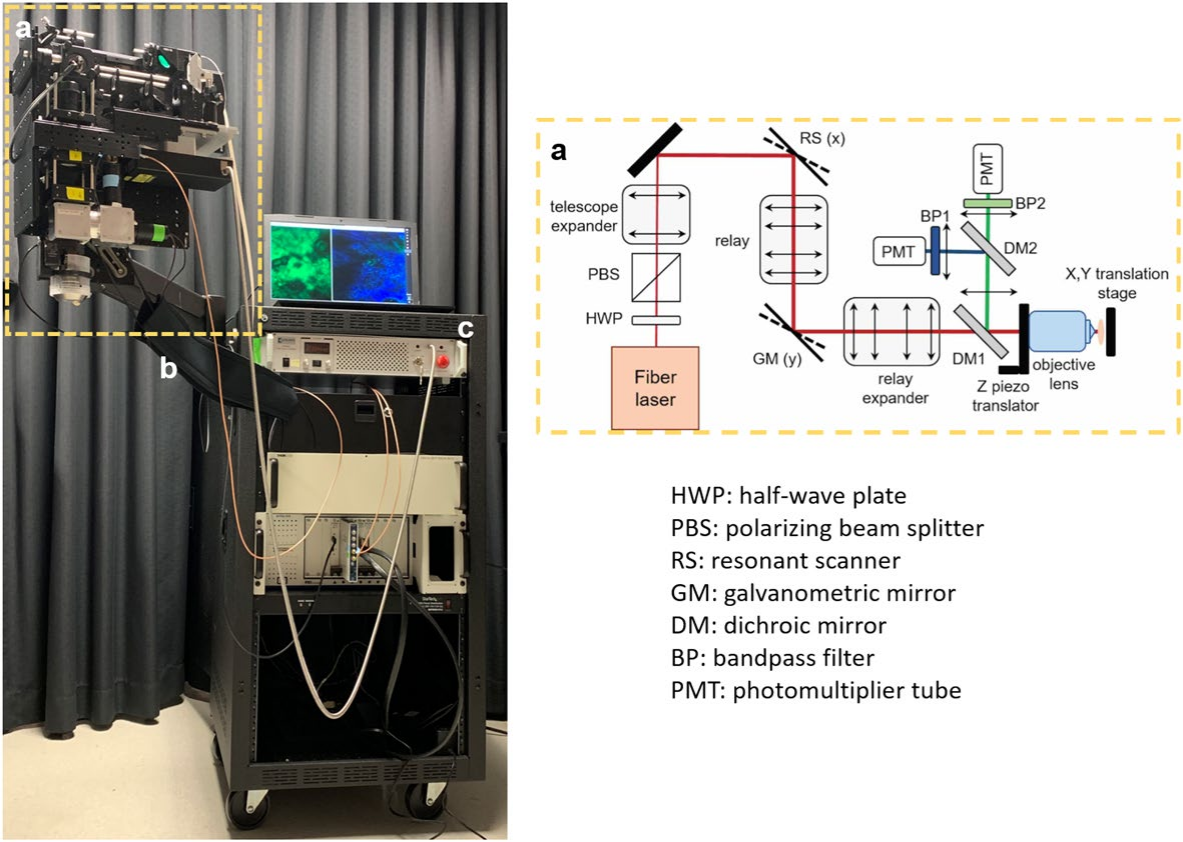
The FLAME system: overview (left) and layout of the scan head (right). The scan head (**a**) is supported by an articulated mechanical arm (**b**) attached to the cart (**c**) that houses the electronic controllers and holds the computer.

The FLAME imaging platform and the layout of its imaging head are presented in Fig. 1. The system is based on a customized optical and opto-mechanical design previously reported by our group^34,39^ and summarized in the “Materials and methods” section. A key component of the current design is related to the excitation light source, a frequency doubled Yb-doped amplified fiber laser (Carmel X-series, Calmar Laser, USA) that generates 90 fs pulses of 780 nm light at 80 MHz and linear p-polarization with a maximum output power of 500 mW. This excitation laser source has replaced the bulky Ti: Sapphire laser (61 × 37 × 19 cm^3^ laser head; 39 × 27 × 39 cm^3^ chiller) employed for excitation in our previously reported MPM platform^34^. The benefit of using this light source is that the laser head has a compact size (9 × 18 × 3.5 cm^3^), which facilitates its incorporation into the imaging scan head (Fig. 1), allowing for enhanced compactness (35 × 35 × 20 cm^3^) and portability of the exoscope.

#### Enhancing imaging speed by utilizing image restoration neural network

**Figure 2:**
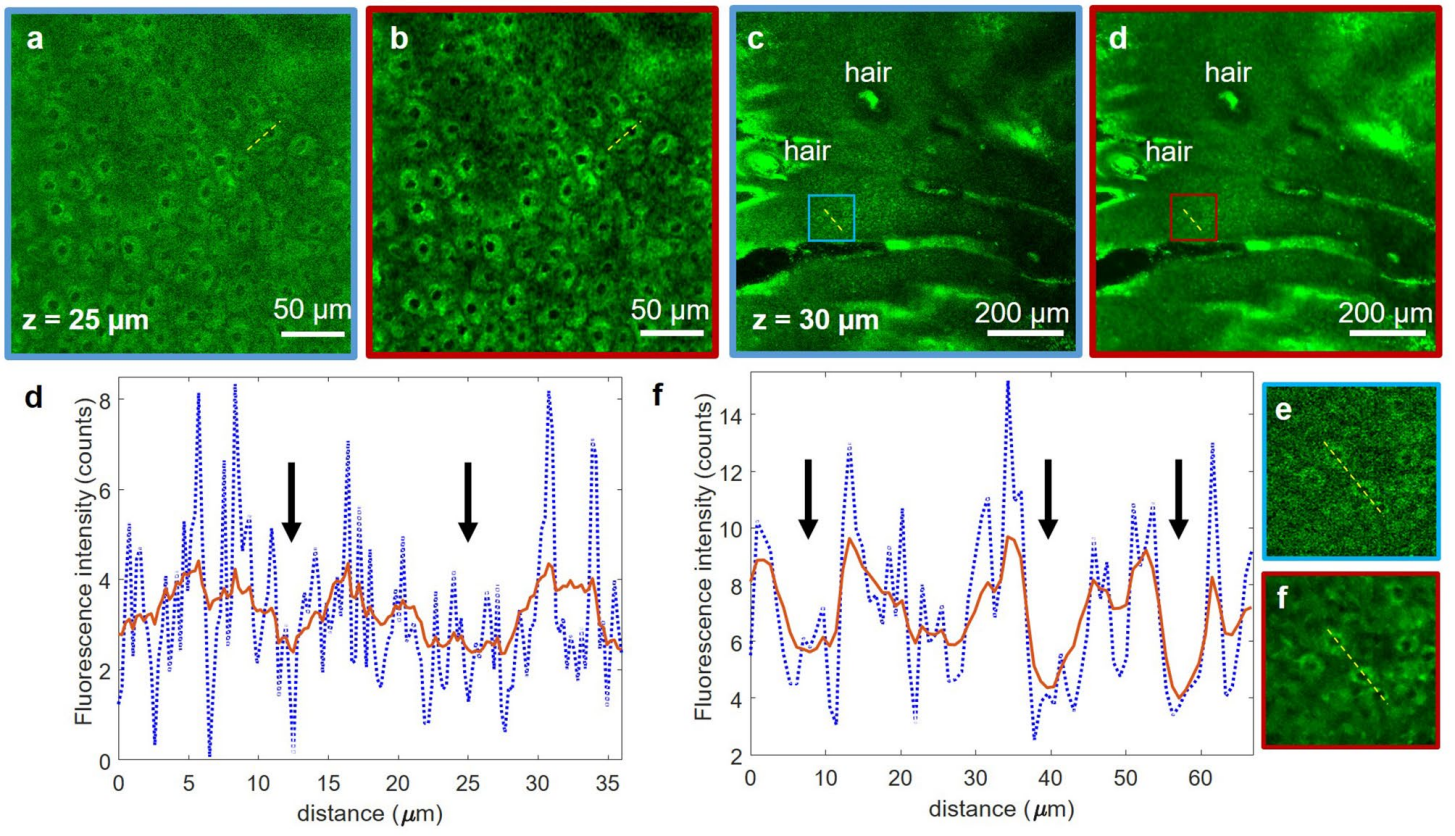
Performance of 2D CARE image restoration. Trained model is applied to images acquired by accumulating 15 consecutive frames of 0.27 × 0.27 mm^2^ (**a**) and 0.9 × 0.9 mm^2^ (**c**) image sequences (both 1 MPx, 2 s acquisition time). Restored images (**b**,**d**) display decreased noise levels and higher contrast for both fields of view. (**e**,**f**) line profiles through adjacent keratinocytes corresponding to (**a**,**b**) and (**c**,**d**) respectively (yellow lines). The line intensity profiles in the network prediction images (red) show enhanced definition of cell nuclei (black arrows) compared to the low cellular contrast to background in the raw images (blue). The insets in (**g**) and (**h**) are close-ups of the blue and red areas outlined in (**c**) and (**d**), respectively.

We accomplish the task of extending the rapid imaging with sub-micron resolution from sub-millimeter to centimeter scale by employing computational approaches along with combined optical and mechanical scanning controlled by optimized data acquisition software. The computational approaches, described in detail in the “Materials and methods” section, involve the use of content-aware image restoration (CARE) in order to compensate for the limited number of photons detected due to the high imaging speed. Figure 2 shows representative examples of TPEF images of keratinocytes in human skin, acquired with the FLAME system and processed with CARE, a neural network model we trained on pairs of TPEF images acquired at fast and slow frame rates in human skin tissue as described in the “Materials and methods” section. The 1 MPx images of 0.27 × 0.27 mm^2^ (Fig. 2a) and 0.9 × 0.9 mm^2^ (Fig. 2c) were acquired by accumulating 15 consecutive frames at an effective rate of 2 s/frame. The corresponding images predicted by the trained CARE network model (Fig. 1b,d) show a significant enhancement of the cellular contrast, allowing for clear delineation of nuclei as shown by the line profiles (Fig. 2e,f) through adjacent keratinocytes in the images of Fig. 2.

The content-aware image restoration has highest impact when applied to multi-millimeter scale mosaic images as it maintains/restores acceptable image contrast while using fewer photons than the current state-of-the-art commercial clinical MPM system, MPTflex by JenLab, due to increase in imaging speed (2 s/mm^2^ vs 96 s/mm^2^, Table S1). This approach provides two key benefits by reducing the acquisition time as well as the risk for potential photodamage by minimizing the exposure time.

#### Rapid, multi-scale, high-resolution imaging by utilizing optical and mechanical scanning along with image restoration

For a comprehensive interrogation of the skin tissue we have devised a multiscale scanning approach that utilizes three complementary scanning modalities (strip mosaic, tile mosaic and volumetric imaging) as described in the “Materials and methods” section. We use these combined optical and mechanical scanning mechanisms in order to mimic the current examination method for histological tissue sections, where the pathologist examines the entire tissue at low magnification and then zooms in to investigate certain regions of interest (ROI).

**Figure 3:**
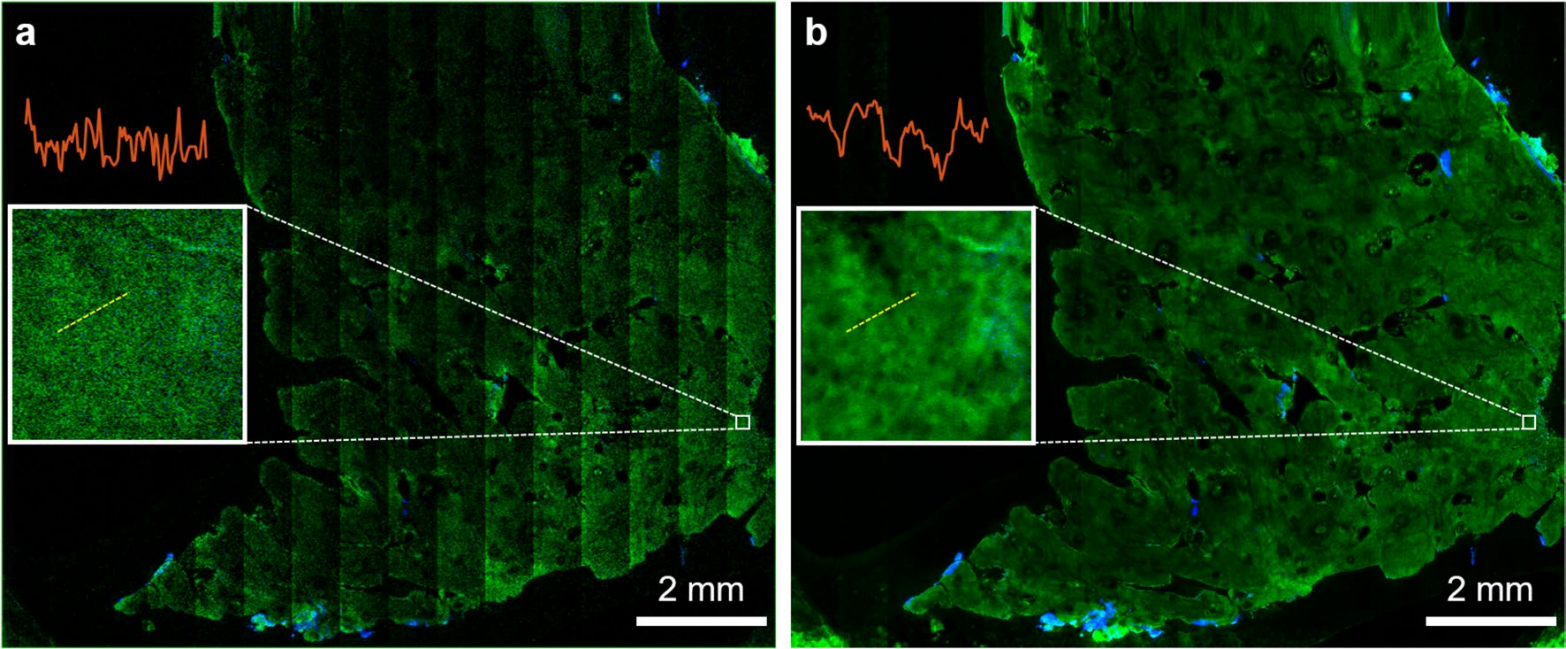
Rapid, centimeter-scale MPM ex vivo imaging of freshly excised facial skin at sub-micron resolution. MPM overview map (80 MPx, 1.2 × 1.0 cm^2^) of a centimeter-scale skin tissue (**a**) prior and (**b**) post CARE processing, acquired as a single frame by strip mosaic scanning at 30 μm depth in the epidermis. The insets represent digital zoom-in of the 200 × 200 μm^2^ outlined areas in (**a**) and (**b**). The intensity profiles (red) through adjacent keratinocytes show that image restoration enhances contrast to allow visualization of keratinocyte nuclei.

Thus, in our imaging procedure, the first step of the tissue examination involves a survey scan on the centimeter scale at about 3 different depths within the epidermis, dermo-epidermal junction (DEJ) and papillary dermis. We employ the single frame strip mosaic scanning mechanism at this spatial scale as it provides significantly faster acquisition rate than the tile mosaic scheme (see “Materials and methods” section). Figure 3 presents a cm-scale MPM image before (Fig. 3a) and after skin-trained CARE network application (Fig. 3b), acquired *ex-vivo* as a single frame at a 30 μm depth within the epidermis of a human skin tissue. The acquisition and restoration time for the image in Fig. 3 (80 MPx, 1.2 × 1.0 cm^2^) was 2 min 15 s. The dynamic range enhancement obtained from neural network restoration allows for trivial field curvature correction (Fig. 3b), not feasible for low SNR raw images (Fig. 3a). The insets represent the digital zoom-in to a 200 × 200 μm^2^ area within the large-scale MPM images, showing that image restoration enhances contrast to allow visualization of keratinocytes nuclei. The slight blur appearing in this image is due to the limited digital resolution the original image was acquired at (1.2 × 1.2 μm^2^ pixel size, see Table S2) and the single frame acquisition rate that results in a low SNR image, sub-optimal for the CARE application model. Nonetheless, the images in Fig. 3 demonstrate that, by using a strip mosaic scanning mechanism and applying a trained neural network model, large areas of skin tissue can be rapidly mapped with sufficiently high resolution and contrast to allow visualization of nuclear and cellular morphology, required to identify ROIs for certain skin conditions.

However, a comprehensive, detailed morphological assessment that allows visualization of microscopic features of interest, such as melanocytic dendrites, requires enhanced resolution and contrast. We accomplish this by zooming in optically and acquire images over millimeter scale tissue areas by using a tile mosaic scanning mechanism. The high resolution and contrast of these mosaic images allow for digitally zooming in to identify architectural features and ROI selection for further volumetric scanning. A movie included in the Supplementary Information (Visualization 1) illustrates the process of navigating a high resolution tile mosaic with digital zoom and lateral browsing highlighting micron-sized melanocytic dendrites that are difficult to visualize in the fully zoomed out images.

**Figure 4:**
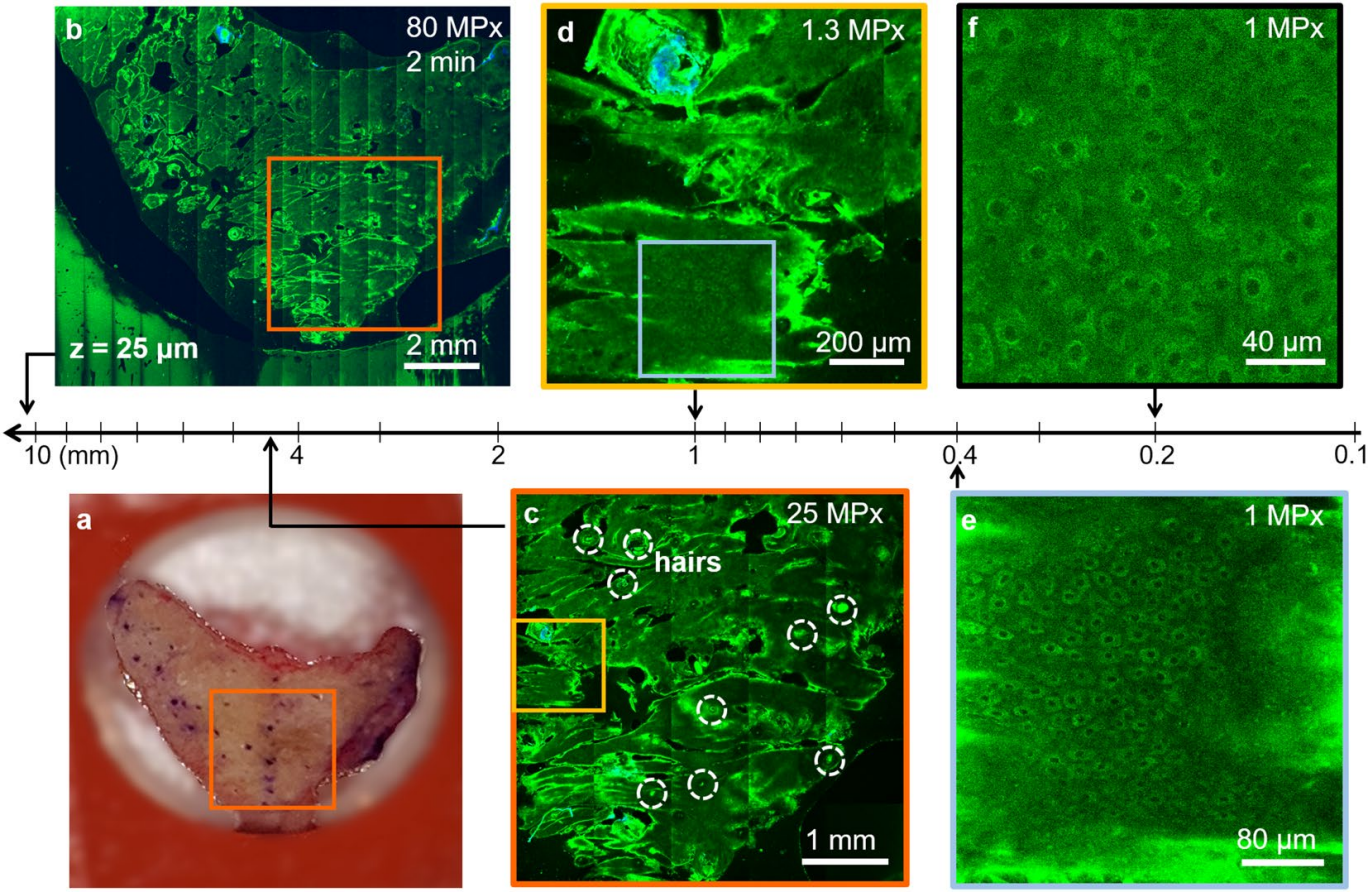
Rapid, multiscale MPM ex vivo imaging of freshly excised facial skin at sub-micron resolution. (**a**) Photographic image of the excised facial skin. (**b**) MPM map overview of the skin tissue epidermis generated by strip-mosaic scanning; the 80 MPx image was acquired at 25 μm depth as a single frame over a 1.2 × 1.0 cm^2^ area and restored with CARE network in 2.15 min. (**c**) Tile mosaic image of the orange outlined area in (**a**) and (**b**) covering 4.5 × 4.5 mm^2^ (25 MPx, acquired and restored with CARE in 2.5 min) with hairs outlined by dashed circles. (**d**) Digital zoom in of the 1 × 1 mm^2^ yellow outlined area in (**c**) showing a close-up of the skin folds, hairs and keratinocytes within the epidermis. (**e**) 1 MPx image acquired in 2 s by optically zooming into the 400 × 400 μm^2^ blue outlined area in (**d**). The image shows well-resolved keratinocytes surrounding a hair follicle. (**f**) Optical zoom in at higher rate sampling 1 MPx, 200 × 200 μm^2^ image allows visualization of intracellular features and protective melanin rings around the nuclei of keratinocytes (200 × 200 μm^2^, 1 MPx image acquired in 2 s).

Figure 4 illustrates the high-resolution, rapid complete mapping of a centimeter to sub-millimeter scale large human skin tissue by combining optical scanning and the two mechanical scanning mechanisms as described in “Materials and methods”, followed by applying the trained CARE neural network. Close-up inspection of the features can be performed by zooming in digitally (Fig. 4d) or optically (Fig. 4e).

Volumetric imaging within selected ROIs is required for in-detail assessment of the morphological changes at different depths. Z-stacks are typically acquired as a sequence of 1 MPx en face images beneath the skin surface, encompassing volumes of about 900 × 900 × 150 μm^2^. A z-stack of 15-accumulated frames for each image, sampling a 900 × 900 × 150 μm^2^ volume every 5 μm, is acquired in 60 s and when needed, restored in 20 s. MPM images acquired ex vivo as a z-stack at different depths in human skin tissue are included in Supplementary Information (Visualization 2).

#### Enhancing molecular image contrast by time-resolved single photon counting (SPC)

**Figure 5:**
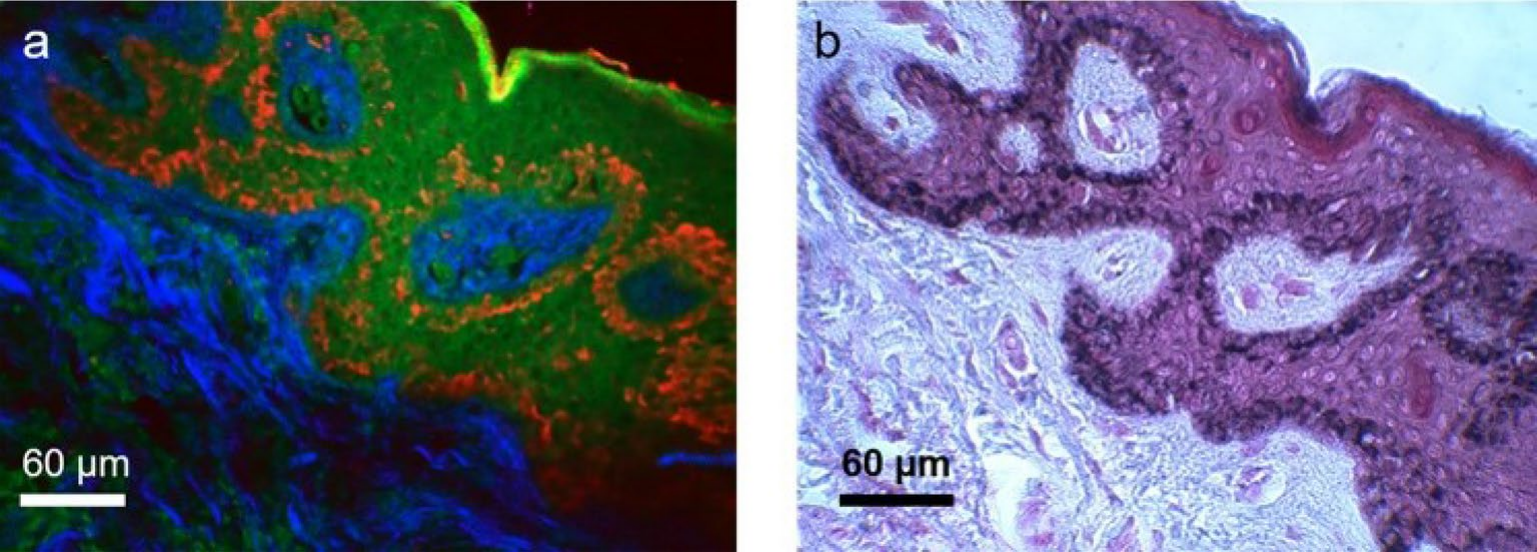
Side-by-side comparison of MPM and histological images. (**a**) time-resolved SPC MPM image of an unstained thin section of formalin-fixed normal human skin acquired with the FLAME system; (**b**) Histological image obtained by Fontana-Masson staining of a human skin section from the same sample and adjacent to the one shown in (**a**). The images show that the fluorescence signal in the corrected-red channel of the MPM image (**a**) is co-localized with the Fontana-Masson melanin-specific stain (black) in the histological image (**b**).

Based on the assignment of the time bin integration in the fluorescence detection channel in our exoscope, as described in “Materials and methods”, melanin is predominantly detected in the corrected red channel, while fluorescence signals from other fluorophores such as keratin, NAD(P)H/FAD and elastin, are mainly detected in the green channel. Figure 5 presents a side-by-side comparison between MPM and histology images of human skin that demonstrate the co-localization of the fluorescence signal in the corrected red channel and the melanin specific stain (Fontana-Masson).

**Figure 6:**
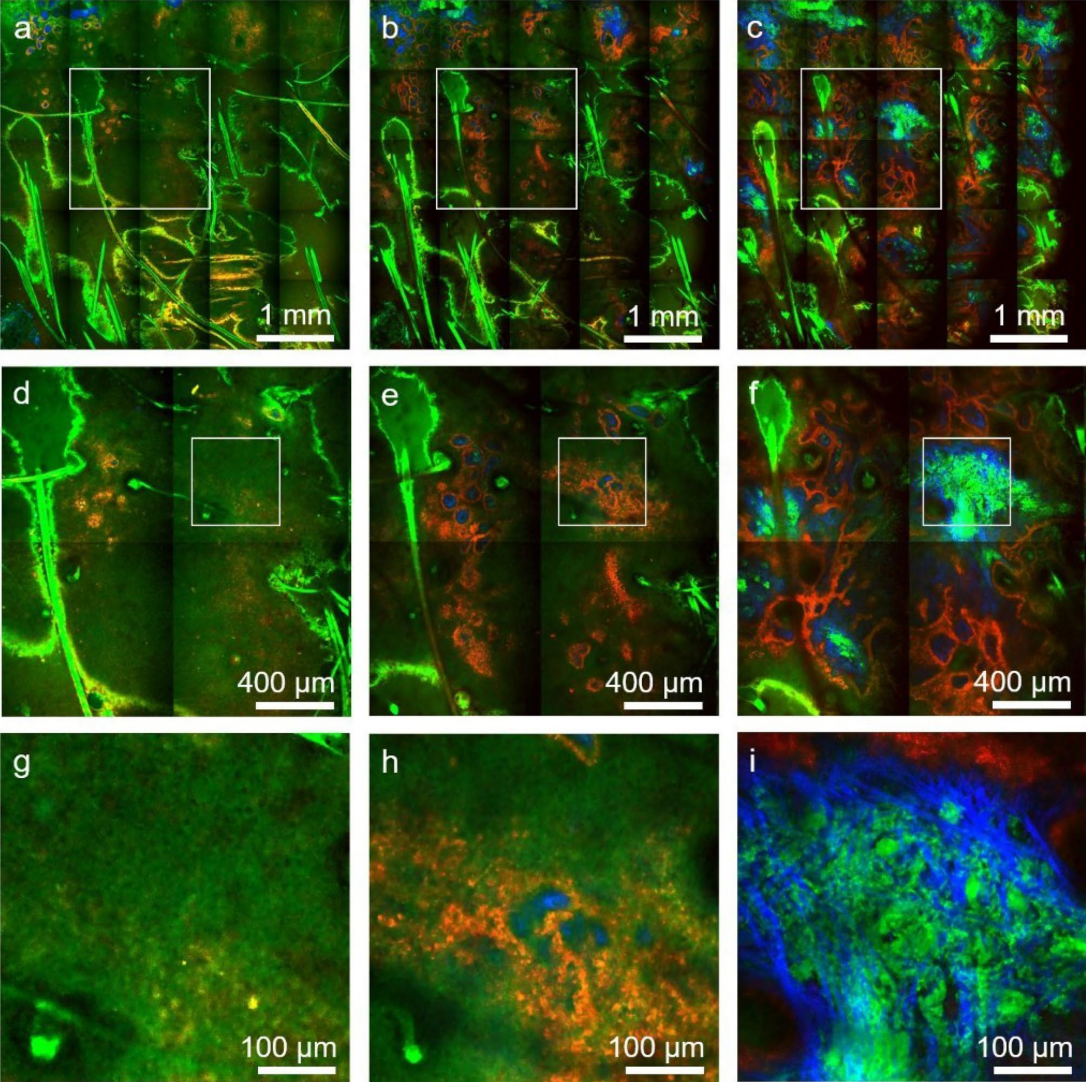
Rapid, millimeter-scale, time-resolved SPC, MPM ex vivo imaging of fresh scalp tissue. Tile-mosaic images (4.5 × 4.5 mm^2^, 25 MPx) acquired and restored with CARE in 2.5 min at select depths of 30 μm (**a**), 50 μm (**b**) and 80 μm (**c**) corresponding to the epidermis, DEJ and papillary dermis, respectively. Mosaic images are color-coded by short-lifetime TPEF (red), long-lifetime TPEF (green) and SHG (blue). (**d**,**e**,**f**) Digital zoom to the 1.8 × 1.8 mm^2^ white outlined areas in (**a**), (**b**) and (**c**), respectively, showing tissue heterogeneity on mm scale. (**g**,**h**,**i**) Digital zoom to the 450 × 450 μm^2^ white outlined areas in (**d**), (**e**) and (**f**), respectively, showing non-pigmented keratinocytes and elastin fibers predominantly visualized in the green channel, pigmented (melanin-rich) keratinocytes mainly detected in the red channel and collagen fibers visualized through the SHG signal in the blue channel.

This approach allows us to enhance the molecular contrast of the FLAME imaging platform without compromising speed. Thus, the time-resolved SPC images and the time-integrated intensity-based images require the same time for acquisition and restoration. Figure 6 illustrates millimeter-scale, sub-micron resolution MPM images based on time-resolved SPC, acquired in a normal pigmented human skin tissue at different depths.

Time-resolved SPC volumetric images acquired *ex-vivo* from pigmented human skin illustrate the ability of the FLAME imaging system to generate 3D distribution maps of melanin (Figure S1, Supplementary Information).

### Rapid multi-scale ex vivo MPM imaging of human skin while maintaining sub-micron resolution

**Figure 7:**
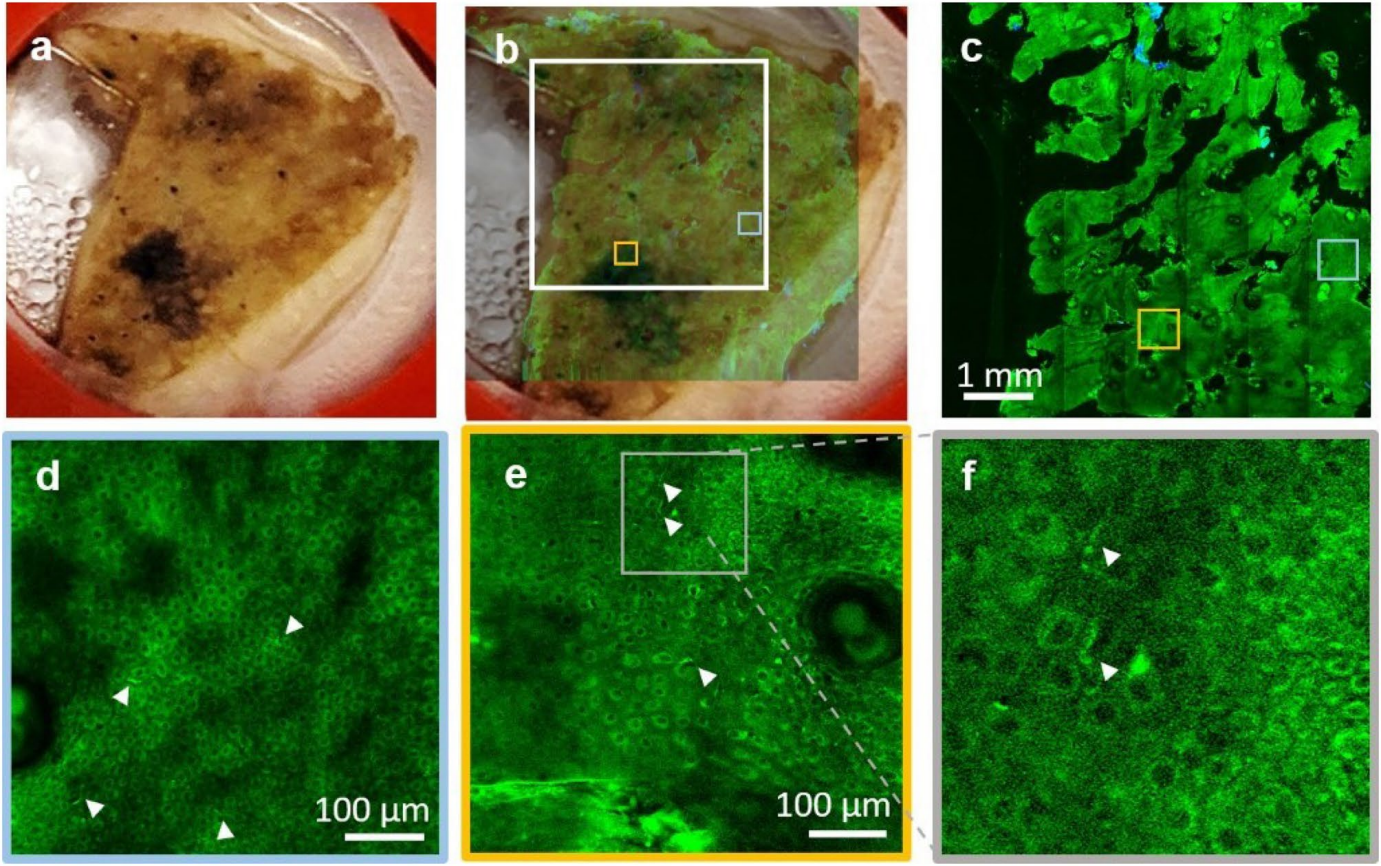
Rapid multi-scale ex vivo MPM imaging with sub-micron resolution to resolve melanocytic dendrites in actinically damaged skin. (**a**) Photographic image of the excised facial skin tissue. (**b**) MPM overview map of the skin tissue epidermis generated by strip-mosaic scanning; the 80 MPx image, acquired as a single frame over a 1.2 × 1.0 cm^2^ area and restored with CARE network in 2.15 min, is illustrated as overlapped with the photographic image of the tissue (opacity 30%). (**c**) High resolution tile mosaic (6.3 × 6.3 mm^2^, 49 MPx) acquired in 3 min with 15 accumulated frames for each tile, at 15 μm below the skin surface. (**d**,**e**) Digital zoom into the blue and yellow outlined locations in (**c**), respectively. Images show the distribution of melanocytic dendrites (arrow heads). (**f**) A close-up of the melanocytic dendrites (white heads) in the outlined area in (**e**). A z-stack acquired at the same location as the inset in (**e**) that shows the 3D appearance of the melanocytic dendrites is included in the Supplementary Information (Visualization 3).

While rapid acquisition of MPM images at millimeter-to-centimeter spatial scale in human skin is critical for enhancing the efficacy and clinical utility of the MPM technology, it is essential to accomplish this goal while maintaining the ability to resolve fine cellular structures. Melanocytic dendrites are a relevant example of fine cellular components that need to be resolved in imaging applications related to melanoma diagnosis, therapy guiding of pigmentary skin disorders or characterizing and understanding of pigment biology. The images in Fig. 7 and in the movie Visualization 1 in Supplementary Information demonstrate the ability of the FLAME imaging system to resolve and rapidly map the spatial distribution of the melanocytic dendrites in the full epidermis of a freshly excised actinically damaged facial skin tissue. A z-stack acquired at the same location as the inset of Fig. 6e shows the 3D appearance of the melanocytic dendrites (Visualization 3, Supplementary Information).

Figure S2 in the Supplementary Information shows an additional example of morphological features which can be identified in human skin lesions based on the sub-micron resolution of the FLAME imaging system. Nests of nevus cells present in a discarded tissue from the excision of a benign compound nevus could be identified in the MPM image and their appearance correlated with histology, as confirmed by a dermatopathologist (R.M.H).

### Rapid millimeter-scale in vivo MPM imaging of human skin at sub-micron resolution

**Figure 8:**
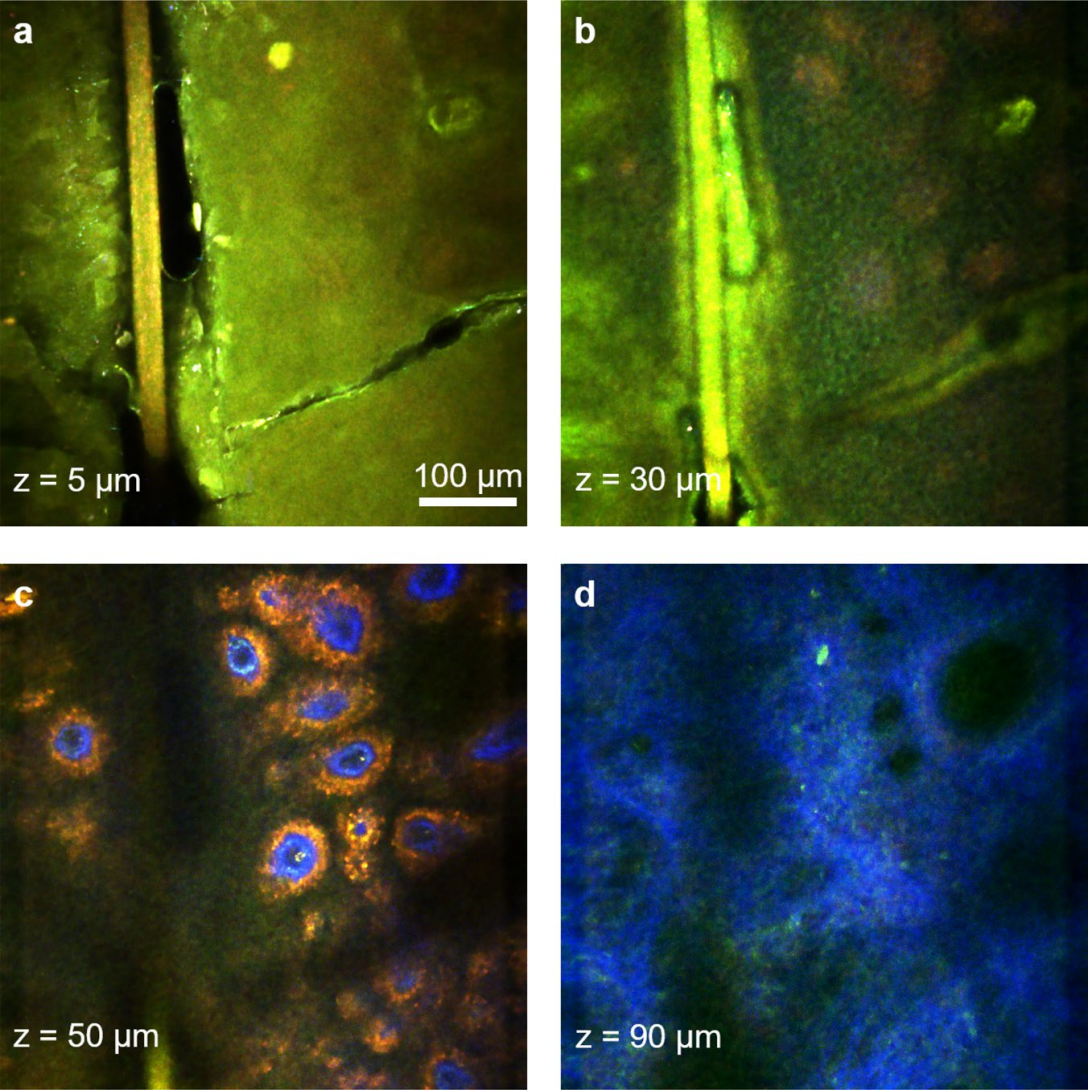
Rapid, sub-micron resolution, time-resolved SPC in vivo MPM imaging of human skin forearm. MPM images acquired at different depths in human skin showing the keratinized stratum corneum (**a**), epidermal keratinocytes (**b**), pigmented cells surrounding dermal papilla (red) (**c**) as well as collagen (blue) and elastin fibers (green) in (**d**).

The ultimate goal in our effort to develop the FLAME imaging platform is to utilize it as an effective imaging tool for in vivo skin imaging in research and clinical applications that require high spatial resolution and molecular contrast. While the imaging system is a bench-top prototype at the current stage, we demonstrate as a proof-of-concept that in vivo imaging using the FLAME platform is within reach. Representative MPM images with time-resolved SPC contrast acquired in vivo at different depths in a subject’s forearm are presented in Fig. 8.

The corresponding full z-stack of 540 × 540 μm^2^ en face images, acquired from the stratum corneum to the papillary dermis with 5 μm axial sampling can be found in the Visualization 4A, Supplementary Information, along with the corresponding 3D melanin distribution (Visualization 4B, Supplementary Information). The time required for acquiring and restoring 30 1 MPx frames within the z-stack was 80 s. A representative millimeter-scale MPM image (2.7 × 2.7 mm^2^) acquired in vivo from a subject’s forearm in 45 s, using time-resolved SPC detection, is included in Supplementary Information (Fig. S3). This is a tile-mosaic image we obtained by using a mechanical component we designed and 3D-printed to connect the miniaturized stage to the subjects’ skin.

## Discussion

In this manuscript, we introduce a compact imaging platform based on label-free multiphoton microscopy optimized for rapid multi-scale imaging of skin tissue, providing up to sub-micron resolution, high contrast images.

One of the major challenges to overcome when performing label-free multiphoton microscopy at high scanning speed is the reduced image contrast as a result of the limited number of detected photons. We address this challenge by implementing a computational approach for image restoration in addition to our previously developed hardware optimization elements. We implemented the recently demonstrated CARE neural network approach^35^, which allowed us to enhance the image contrast and extend the scanning area to centimeter scale. Although scanning area and speed are independent parameters, they are closely related in the context of clinical translation of the MPM technology. Imaging of large tissue areas is only valuable and feasible in clinical setting when performed at high scanning rates to minimize motion artifacts and optimize clinical work-flow. We used fast, miniature linear stages and custom data acquisition software design to integrate a strip-mosaic scanning scheme. This approach, in addition to our existing tile-mosaic and volumetric imaging mechanisms, extended the spatial scanning range, allowing for rapid imaging of sub-millimeter to centimeter scale areas. A similar approach has been previously described and implemented as a solution for rapid reflectance confocal imaging, although with a much larger stage suitable for ex vivo imaging^40,41^.

An additional challenge to overcome in multiphoton microscopy is maintaining high spatial resolution while optimizing scanning area and speed. The high numerical aperture objective and the custom optical design of our imaging platform provide millimeter-scale images at sub-micron resolution^34^. The centimeter-scale areas are scanned rapidly at micron resolution, sufficiently high to allow, in concert with the image contrast enhancement provided by the CARE neural network model, visualization of the cellular and fibrillar structures. This performance element is critical in the ability of the exoscope to emulate the current histological examination method, where the pathologist examines the entire tissue at low magnification and then zooms in for careful investigation of selected regions of interest. Although for the work described in this manuscript, the CARE model was trained and applied mainly on normal skin tissue samples, we expect the model to perform well on various skin conditions. While these are characterized by different architectural patterns, the main skin features used for training are the same and the variance in SNR is expected to be influenced mainly by the amount of pigmentation characteristic to the particular skin conditions (melanin is the brightest fluorophore in skin tissue). We have trained the CARE model on skin tissue samples with different degrees of pigmentation (Fitzpatrick skin types I–III).

Molecular contrast is the key benefit of MPM when compared to high-resolution imaging techniques such as reflectance confocal microscopy or optical coherence tomography, currently utilized for clinical skin imaging. The molecular contrast, commonly provided in MPM skin imaging by the intensity of the endogenous fluorescence signals, can be significantly enhanced through approaches such as, fluorescence lifetime imaging (FLIM). FLIM has been demonstrated to be valuable for many applications related to label-free skin imaging in humans^42^ and animal models^43^. However, the conventional approach, time-correlated single photon counting (TCSPC), although it enables acquisition of fluorescence lifetime decays with high temporal resolution, for fluorophores in skin hundreds of photons per pixel^43^ are required for accurate lifetime decay curve representation, which leads to long integration times. TCSPC imaging of skin in humans and animal models has been commonly performed at 250 ps temporal resolution and 25 s^42^ to 60 s^43^ integration time per 256 × 256 pixel frame. These values would translate into hundreds of minutes acquisition time for megapixel size images as required for imaging millimeter-to-centimeter skin tissue areas. One solution we found for enhancing the intensity-based molecular contrast of the FLAME imaging platform without compromising speed, was to integrate time resolved SPC detection with reduced temporal resolution (780 ps) and time bins selected and grouped in such a way to allow selective discrimination of just one key endogenous fluorophore in skin, melanin. This approach is facilitated by the characteristically short fluorescence lifetime of a melanin component^42,44,45^, probably eumelanin^10^, with respect to the other endogenous fluorophores in skin. Selective detection of melanin is essential in distinguishing dermal melanocytes and melanophages from other cellular components and in applications such as pigmented lesions diagnosis and therapy assessment of pigmentary skin disorders requiring quantitative assessment of melanin.

A key element of the imaging system design is the excitation light source, a compact femtosecond fiber laser that allows its integration into the exoscope imaging head, significantly enhancing the instrument compactness and facilitating its conversion to a portable device.

We demonstrated the exoscope performance by ex vivo and in vivo imaging of human skin. The FLAME platform can provide millimeter to centimeter scale high contrast images of skin in about 2 min with sub-micron and micron resolution, respectively. Depth-resolved images over volumes of 900 × 900 × 150 μm^3^ are acquired in 1 min. The spatial resolution performance was demonstrated by the ability of the exoscope to visualize melanocytic dendrites in actinically damaged human skin. Melanocytes are identified in MPM imaging by their dendritic presence. If the melanocytic dendrites are not resolved, melanocytes very often cannot be distinguished from pigmented keratinocytes. Identifying the melanocytic presence is essential in applications such as melanoma diagnosis, therapy guiding of pigmentary skin disorders or characterizing and understanding of pigment biology. An additional benefit of using FLAME as an imaging tool for these applications is related to its unique ability to provide in real-time, a volumetric mapping of melanin as demonstrated by the images we acquired ex vivo and in vivo in human skin. Rapid access to a 3D map of melanin distribution in skin is expected to expedite and enhance the accuracy of its quantitative assessment, a particularly important tool for diagnosis and therapy guiding of pigmentary skin disorders^13^ and for characterizing and understanding of pigment biology^22^.

While these features represent a significant enhancement of the scanning area and speed, molecular contrast and the overall compactness of the FLAME imaging platform, limited penetration depth in scattering tissues such as skin, still remains. Penetration depth can be optimized by using a longer excitation wavelength^46–48^, by compensating for dispersion to maintain the laser pulse duration within the tissue^49^ or by adaptive optical correction of aberrations to recover diffraction-limited performance at depth in tissue^50^. Despite this limitation, the current penetration depth of 150–200 μm is sufficient to capture early signs of malignancy in skin that occur at the DEJ^14,17,19^, to detect the presence of dermal melanocytes^14^ and to identify existing or prior inflammatory response in the papillary dermis^13,21^.

In conclusion, we introduce a compact, multiphoton microscopy-based imaging platform, FLAME, highly optimized for rapid, label-free macroscopic imaging of skin with microscopic resolution and high contrast. It has the ability to provide 3D images encompassing sub-millimeter to centimeter scale areas of skin tissue within minutes. It allows fast discrimination and 3D virtual staining of melanin. This unique combination of features provides the FLAME imaging platform with highly optimized functionality and facilitates a seamless conversion to a portable device for rapid, multi-scale clinical skin imaging with high resolution.

## Materials and methods

### MPM imaging platform

The optical layout as presented in Fig. 1 includes a 4 kHz resonant-galvo scanning mechanism, custom-designed relay and beam expander optics and a 25 × , 1.05 NA objective lens (XLPL25XWMP, Olympus). The optical design of this system was optimized to provide sub-micron resolution images over a field-of-view of 0.8 × 0.8 mm^2^ at speeds of less than 2 s per 1 MPx frame for high SNR ^34^. The low image contrast caused by the limited number of detected photons due to high scanning speed was maximized by employing sensitive PMTs (Hamamatsu R9880-20 and R9880-210) in photon counting mode and by optimizing the signal collection optics to capture the maximum amount of radiation emitted in the epi-direction^34^. We use a first dichroic mirror to separate the excitation and the emission signals (FF705-Di01, Semrock, Inc.) and a second dichroic mirror (FF506-Di03, Semrock, Inc.) to split the TPEF and SHG detection channels defined by the emissions filters: FF01-720/SP and FF01-535/150 (Semrock, Inc.) for TPEF; and FF01-375/110 for SHG. These optical elements allow the detection of the SHG signal from collagen and the TPEF signals from NAD(P)H/FAD, keratin, melanin and elastin in skin^8,51,52^.

The mechanical scanning for rapid movement of the skin tissue is performed by fast, miniature linear xy stages (Q522.130, Physik Instrumente, GmbH) controlled by a customized data acquisition software (Vidrio Technologies), which synchronizes their travel with the laser beam scanning to facilitate rapid stitching of adjacent scanned areas. We selected this stage based on its performance and its reduced size (3 × 2.1 × 1 cm^3^), the latter being an essential requirement for its integration into the compact scanning head. The stage can be used as the mechanical scanner component while imaging either ex vivo or in vivo by taking advantage of the skin elasticity. The approach of combining the mechanical and optical scanning to rapidly image sub-millimeter to centimeter scale tissue areas is described in “Multi-scale, high-resolution imaging by optical and mechanical scanning” section.

The data acquisition software and hardware are critical components that affect the performance of the exoscope. Our current design employs the Matlab based software ScanImage (Vidrio Technologies) and data acquisition (DAQ) National Instruments (NI) hardware based on a fast digitizer (NI 5771) controlled by a FlexRIO FPGA module (NI PXIe-7975). The software enables single photon counting (SPC) detection and time-resolved SPC with a ~ 780 ps (16 bins) temporal resolution by using the 80 MHz laser sync signal, upmultiplied 16 × with a clock generator (AD9516-0, Mouser), as an external clock for the fast digitizer. Given the rise time of the PMT (R9880U-20) of 570 ps the temporal resolution is sampling-limited. Hence, detected photons may be binned into virtual output channels based on their arrival time, allowing for selective detection of some fluorophores. Thus, for time-resolved SPC imaging, the signal was detected based on the fluorescence photons arrival time relative to the excitation photons, in time bins of width: -0.4–1.2 ns (red channel) and 0.4–12 ns (green channel). The rationale for defining these time bins was for attaining selective detection of melanin. The fluorescence of melanin, is characterized by short lifetime with respect to most other endogenous fluorophores in skin^42,44,45^. Due to the integration time bin overlap of the two detection channels and the modest temporal resolution, subtraction of their intensity (photon counts) signals is required to obtain a differential signature of melanin. Thus, we subtract the photon counts representing the long lifetime fluorescence signal (green channel image) from the short lifetime fluorescence signal (red channel image). The subtraction process yields both positive and negative values. The negative values are due to larger contribution to signal from photons with long fluorescence lifetime. These values are set to zero in the final image resulted from subtraction. The positive photon count values resulted from subtraction are due to larger contribution to signal from photons with short fluorescence lifetime. These values represent mainly the melanin fluorescence signal contribution and the remaining signal in the final image (corrected-red channel) resulted from subtraction. We perform the time-resolved SPC volumetric imaging using 3 channels for simultaneous detection of the SHG signal and of the TPEF signals characterized by the short and long fluorescence lifetime as described above. The 3D distribution of melanin is available almost instantaneous after correcting for signal overlap.

### Laser power considerations to avoid potential photodamage

Besides minimizing exposure time through rapid scanning, we also ensure the laser fluence at the sample is below the established thermal^53^ and DNA^54^ damage threshold for two-photon microscopy of human skin. Thus, based on the focusing optics in our exoscope (NA = 1.05) and the 45 mW excitation laser power at the skin surface, the laser fluence value is lower by at least a factor of 1.7 compared to the fluence values the CE-certified clinical MPM device (MPTflex, JenLab) employs at the skin surface and the fluence values for DNA and thermal damage threshold established for two-photon microscopy of human skin^53,54^.

### Neural network training

We use content-aware image restoration (CARE)^35^, recently described as an effective image denoising technique, to restore fluorescence microscopy images acquired with fewer photons for enhancing imaging speed and limit light exposure time. In this method, pairs of images are acquired at low and high SNR for training a sample-specific convolutional neural network by using the high SNR images as ground truth (Supplemental Figure S4). The trained network is then applied to restore low SNR images to predict the high SNR output. The high SNR images, serving as ground truth for training the model, were obtained by accumulating 70 consecutive frames. This number of accumulated frames corresponds to ~ 9 μs effective pixel dwell time, which results in a signal level sufficiently high to cover an 8-bit dynamic range. The model was trained with two inputs—7 and 15 accumulated frames. Using two inputs allowed us to account for two different SNR values generally characteristic to different levels of pigmentation (melanin is the brightest fluorophore in skin with MPM imaging and thus, the amount of pigmentation is generally closely correlated with the image SNR). The two inputs approach was also found to provide robustness to the network performance as described in the original publication by the developers of CARE^35^. The reason for selecting the values of 7 and 15 as the numbers of accumulated frames for the two inputs was related to practical considerations regarding the need to minimize of imaging speed. The 7 and 15 accumulated frames values correspond to ~ 1 and 2 μs pixel dwell time that results in ~ 1 and 2 min acquisition time, respectively for 20 mm^2^ 5120 × 5120 pixel mosaics. Based on our group’s long time experience in clinical skin imaging with MPM, these time frames would be considered acceptable in clinical setting.

Our training set consisted of 128 × 128 pixel patches (112 × 112 μm^2^) extracted from 1024 × 1024 pixel images (900 × 900 μm^2^). The patch size was selected based on the minimum amount of area required to avoid overtraining the model while covering features in skin with different spatial scales, such as cellular features (keratinocytes, melanocytes, corneocytes) on the order of 5–10 μm and tissue features (hairs, skin folds, sweat glands) on average < 100 μm. Quantification of model performance on a validation set with mean standard error revealed minor improvement increasing the training patch size from 64 × 64 pixels to 128 × 128 pixels and no observable difference for 256 × 256 pixels.

The network was trained on 550 image pairs generated by ex vivo imaging of freshly excised human skin tissue (8 excisions including pigmented and nonpigmented skin) at different depths within viable epidermis. CARE model was trained on a diverse set of features such as corneocytes, keratinocytes, melanin caps, hair follicles, sweat glands, elastin fibers and skin folds. The training set was acquired by scanning 20 mm^2^ over 6–10 optical sections at different depths spaced out by 10 μm spanning from skin surface to the papillary dermis. This volumetric imaging procedure ensured a fair representation of skin features accessible within the depth limitation of noninvasive MPM in vivo imaging (< 300 μm from the skin surface). We used a total of 35,200 patches for the network training pooled together from all the specimens. Ten percent of these images (3,520) were randomly selected for the validation set, while the remaining 31,680 images were used for training. We applied this approach only to the TPEF images acquired by the FLAME system, as the SHG signal was sufficiently high at fast scanning rates. Training the model over 100 epochs with 30 steps per epoch resulted in a mean square error of 0.005 and a mean absolute error of 0.05 on the validation set. On an independent test set consisting of 48 900 × 900 µm^2^ 1 MPx images from 3 subjects CARE-processed images show an increase in structural similarity (SSIM) index (0.6–0.7) relative to the high SNR ground truth (70 accumulations) compared to low SNR (15 accumulations) input images (0.3–0.5). Representative input-ground truth image pairs from the test set along with the output image generated by the model are shown in Figure S5 in the Supplementary Information.

### Multi-scale, high-resolution imaging by optical and mechanical scanning

We perform imaging on millimeters to centimeter scale by employing the following scanning mechanisms for stitching adjacent fields of views and for acquiring volumetric images:

1. *the strip mosaic scheme* employs the resonant galvo mirror as the fast scanner in the x direction and a miniature linear stage as the slow scanner in the y direction. The width of the strip is defined by the laser line produced by the fast scanning mirror, while its length is determined by the stage travel range. The stage moves back in the –y direction and also laterally in the x direction, a distance equal with the width of the strip, before completing each of the subsequent strips. We commonly acquire 10 mm long, 0.75 mm wide strips, resulting in an aspect ratio of 1:13. Depending on the sample size, we acquire up to 16 strips resulting in a scanned area of 10 × 12 mm^2^, typically collected as 64 MPx image and rebinned using bicubic interpolation to maintain the correct aspect ratio to 80 MPx. The acquisition and restoration time for this type of image is 2 min 15 s. The length and the number of the strips are limited by the stage speed, its travel range (13 mm) and its synchronization with the resonant scanner.
2. *the tile mosaic scheme* employs the galvo-resonant mirrors to scan each frame (‘tile’) and the linear stages to translate the sample in the x and y directions in order to scan adjacent tiles.
3. *the volumetric imaging* involves acquiring z-stacks of en face images by moving the objective in the z direction, thus scanning at different depths in the skin.

Strip mosaic provides the fastest scanning rate for single frame acquisition. We employ the single frame strip mosaic scanning mechanism for centimeter spatial scale imaging. However, multiple frames accumulation is often required at millimeter-scale FOV for enhanced contrast to allow accurate morphological assessment. In this case, our current data acquisition software provides a faster scanning rate for the tile mosaic configuration. At millimeter scale, we commonly acquire tile mosaic images at 15-accumulating frames. Figure S6 in the Supplementary Information presents a comparison of the tile and strip mosaic scanning speed for different configurations we typically employ for the multi-scale imaging. Table S2 in the Supplementary Information summarizes the parameters (digital resolution and scanning time) used for scanning different spatial scales.

### Excised tissue collection and in vivo imaging

Skin specimens were obtained from the surgeries in the UCI Dermatology Clinic and consisted of remaining tissue from the wound closure procedures (“dog ears”). The tissue collection procedure was exempt from the Institutional Review Board (IRB) approval since the specimens were de-identified, but they were handled based on an Institutional Biosafety Committee (IBC) approved protocol. The specimens were imaged fresh, immediately upon collection. The in vivo measurements were conducted according to an approved IRB protocol of the University of California–Irvine with written informed consent obtained from the subjects.

## Supplementary information

Supplementary Video 1.

Supplementary Video 2.

Supplementary Video 3.

Supplementary Video 4A.

Supplementary Video 4B.

Supplementary Information 1.

## Data Availability

The datasets generated and analyzed in the article are available from the corresponding author upon reasonable request.

## References

[1] Guitera P (2009). In vivo reflectance confocal microscopy enhances secondary evaluation of melanocytic lesions. J. Investig. Dermatol..

[2] Langley RG (2007). The diagnostic accuracy of in vivo confocal scanning laser microscopy compared to dermoscopy of benign and malignant melanocytic lesions: a prospective study. Dermatology.

[3] Gonzalez S, Sanchez V, Gonzalez-Rodriguez A, Parrado C, Ullrich M (2014). Confocal microscopy patterns in nonmelanoma skin cancer and clinical applications. Actas dermo-sifiliograficas.

[4] Nori S (2004). Sensitivity and specificity of reflectance-mode confocal microscopy for in vivo diagnosis of basal cell carcinoma: a multicenter study. J. Am. Acad. Dermatol..

[5] Gambichler T (2007). In vivo optical coherence tomography of basal cell carcinoma. J. Dermatol. Sci..

[6] Olmedo JM, Warschaw KE, Schmitt JM, Swanson DL (2006). Optical coherence tomography for the characterization of basal cell carcinoma in vivo: a pilot study. J. Am. Acad. Dermatol..

[7] Masters BR, So PT, Gratton E (1997). Multiphoton excitation fluorescence microscopy and spectroscopy of in vivo human skin. Biophys. J..

[8] Konig K, Riemann I (2003). High-resolution multiphoton tomography of human skin with subcellular spatial resolution and picosecond time resolution. J. Biomed. Opt..

[9] Breunig HG, Studier H, Konig K (2010). Multiphoton excitation characteristics of cellular fluorophores of human skin in vivo. Opt. Express.

[10] Krasieva TB (2013). Two-photon excited fluorescence lifetime imaging and spectroscopy of melanins in vitro and in vivo. J. Biomed. Opt..

[11] Balu M (2013). In vivo multiphoton NADH fluorescence reveals depth-dependent keratinocyte metabolism in human skin. Biophys. J..

[12] Pouli D (2016). Imaging mitochondrial dynamics in human skin reveals depth-dependent hypoxia and malignant potential for diagnosis. Sci. Transl. Med..

[13] Lentsch G (2019). In vivo multiphoton microscopy of melasma. Pigment Cell Melanoma Res..

[14] Balu M (2014). Distinguishing between benign and malignant melanocytic nevi by in vivo multiphoton microscopy. Cancer Res..

[15] Puschmann S, Rahn CD, Wenck H, Gallinat S, Fischer F (2012). Approach to quantify human dermal skin aging using multiphoton laser scanning microscopy. J. Biomed. Opt..

[16] Lin SJ (2005). Evaluating cutaneous photoaging by use of multiphoton fluorescence and second-harmonic generation microscopy. Opt. Lett..

[17] Dimitrow E (2009). Sensitivity and specificity of multiphoton laser tomography for in vivo and ex vivo diagnosis of malignant melanoma. J. Investig. Dermatol..

[18] Paoli J, Smedh M, Wennberg AM, Ericson MB (2008). Multiphoton laser scanning microscopy on non-melanoma skin cancer: morphologic features for future non-invasive diagnostics. J. Investig. Dermatol..

[19] Balu M (2015). In vivo multiphoton microscopy of basal cell carcinoma. JAMA Dermatol..

[20] Lentsch, G. *et al.* Non-invasive optical biopsy by multiphoton microscopy identifies the live morphology of common melanocytic nevi. *Pigment Cell and Melanoma Res. ***33, **869-877. https://doi.org/10.1111/pcmr.12902 (2020). 10.1111/pcmr.12902 PMC7687135 32485062

[21] Lin J (2019). Feature characterization of scarring and non-scarring types of alopecia by multiphoton microscopy. Lasers Surg. Med..

[22] Saager RB (2015). In vivo measurements of cutaneous melanin across spatial scales: using multiphoton microscopy and spatial frequency domain spectroscopy. J. Biomed. Opt..

[23] Balu M (2017). In vivo multiphoton-microscopy of picosecond-laser-induced optical breakdown in human skin. Lasers Surg. Med..

[24] Bevona C, Goggins W, Quinn T, Fullerton J, Tsao H (2003). Cutaneous melanomas associated with nevi. Arch. Dermatol..

[25] Cahill LC (2019). Comparing histologic evaluation of prostate tissue using nonlinear microscopy and paraffin H&E: a pilot study. Mod. Pathol..

[26] Giacomelli MG (2019). Comparison of nonlinear microscopy and frozen section histology for imaging of Mohs surgical margins. Biomed. Opt. Express.

[27] Giacomelli MG (2016). Virtual hematoxylin and eosin transillumination microscopy using epi-fluorescence imaging. PLoS ONE.

[28] Chang H (2020). Moxifloxacin labeling-based multiphoton microscopy of skin cancers in Asians. Lasers Surg. Med..

[29] Tsai PS (2015). Ultra-large field-of-view two-photon microscopy. Opt. Express.

[30] Terada SI, Kobayashi K, Ohkura M, Nakai J, Matsuzaki M (2018). Super-wide-field two-photon imaging with a micro-optical device moving in post-objective space. Nat. Commun..

[31] Abdeladim L (2019). Multicolor multiscale brain imaging with chromatic multiphoton serial microscopy. Nat. Commun..

[32] Stirman JN, Smith IT, Kudenov MW, Smith SL (2016). Wide field-of-view, multi-region, two-photon imaging of neuronal activity in the mammalian brain. Nat. Biotechnol..

[33] Sofroniew NJ, Flickinger D, King J, Svoboda K (2016). A large field of view two-photon mesoscope with subcellular resolution for in vivo imaging. Elife.

[34] Balu M, Mikami H, Hou J, Potma EO, Tromberg BJ (2016). Rapid mesoscale multiphoton microscopy of human skin. Biomed. Opt. Express.

[35] Weigert M (2018). Content-aware image restoration: pushing the limits of fluorescence microscopy. Nat. Methods.

[36] Manifold B, Thomas E, Francis AT, Hill AH, Fu D (2019). Denoising of stimulated Raman scattering microscopy images via deep learning. Biomed. Opt. Express.

[37] Laine, S., Karras, T., High-Quality Self-Supervised Deep Image Denoising Lehtinen, J. & Aila, T. High-Quality Self-Supervised Deep Image Denoising. (2019) https://arxiv.org/abs/1901.10277.

[38] Tian C (2020). Deep learning on image denoising: an overview. Neural Netw..

[39] Balu, M., Potma, E. O., Tromberg, B. J. & Mikami, H. Imaging platform based on nonlinear optical microscopy for rapid scanning large areas of tissue US patent 10,595,770 B2 (2020).

[40] Abeytunge S (2013). Confocal microscopy with strip mosaicing for rapid imaging over large areas of excised tissue. J. Biomed. Opt..

[41] Abeytunge S, Li Y, Larson B, Toledo-Crow R, Rajadhyaksha M (2011). Rapid confocal imaging of large areas of excised tissue with strip mosaicing. J. Biomed. Opt..

[42] Seidenari S (2013). Multiphoton laser tomography and fluorescence lifetime imaging of melanoma: morphologic features and quantitative data for sensitive and specific non-invasive diagnostics. PLoS ONE.

[43] Skala MC (2007). In vivo multiphoton microscopy of NADH and FAD redox states, fluorescence lifetimes, and cellular morphology in precancerous epithelia. Proc. Natl. Acad. Sci. U. S. A..

[44] Dancik Y, Favre A, Loy CJ, Zvyagin AV, Roberts MS (2013). Use of multiphoton tomography and fluorescence lifetime imaging to investigate skin pigmentation in vivo. J. Biomed. Opt..

[45] Dimitrow E (2009). Spectral fluorescence lifetime detection and selective melanin imaging by multiphoton laser tomography for melanoma diagnosis. Exp. Dermatol..

[46] Balu M (2009). Effect of excitation wavelength on penetration depth in nonlinear optical microscopy of turbid media. J. Biomed. Opt..

[47] Kobat D (2009). Deep tissue multiphoton microscopy using longer wavelength excitation. Opt. Express.

[48] Balu M, Saytashev I, Hou J, Dantus M, Tromberg BJ (2015). Sub-40 fs, 1060-nm Yb-fiber laser enhances penetration depth in nonlinear optical microscopy of human skin. J. Biomed. Opt..

[49] Tang S, Krasieva TB, Chen Z, Tempea G, Tromberg BJ (2006). Effect of pulse duration on two-photon excited fluorescence and second harmonic generation in nonlinear optical microscopy. J. Biomed. Opt..

[50] Wang K (2015). Direct wavefront sensing for high-resolution in vivo imaging in scattering tissue. Nat. Commun..

[51] Pena A, Strupler M, Boulesteix T, Schanne-Klein M (2005). Spectroscopic analysis of keratin endogenous signal for skin multiphoton microscopy. Opt. Express.

[52] Zoumi A, Lu X, Kassab GS, Tromberg BJ (2004). Imaging coronary artery microstructure using second-harmonic and two-photon fluorescence microscopy. Biophys. J..

[53] Masters BR (2004). Mitigating thermal mechanical damage potential during two-photon dermal imaging. J. Biomed. Opt..

[54] Fischer F (2008). Risk estimation of skin damage due to ultrashort pulsed, focused near-infrared laser irradiation at 800 nm. J Biomed. Opt..

