# Supplementary material for: Fast, large area multiphoton exoscope (FLAME) for macroscopic imaging with microscopic resolution of human skin
Source: Sci Rep. 2020 Oct 22;10:18093. doi: 10.1038/s41598-020-75172-9 (PMC7582965; doi:10.1038/s41598-020-75172-9)
Supplement: Supplementary file 6 — Supplementary Information 1. [file 41598_2020_75172_MOESM6_ESM.pdf]

Fast, large area multiphoton exoscope (FLAME) for macroscopic imaging with microscopic resolution of human skin

Alexander Fast[1], Akarsh Lal[1], Amanda F. Durkin[1], Griffin Lentsch[1], Ronald M. Harris[2], Christopher B. Zachary[2], Anand K. Ganesan[2], Mihaela Balu[1,*]

[1] Beckman Laser Institute and Medical Clinic, University of California, Irvine, 1002 Health Sciences Rd., Irvine, California, 92612

[2] Department of Dermatology, University of California, Irvine, 1 Medical Plaza Dr., Irvine, California, 92697

[*]mbalu@uci.edu

Authors' Contact Information

Alexander Fast, afast@uci.edu

Akarsh Lal, akarshl@uci.edu

Amanda F. Durkin, afdurkin@uci.edu

Griffin Lentsch, glentsch@uci.edu

Ronald M. Harris, rmharris@uci.edu

Christopher B. Zachary, czachary@uci.edu

Anand K. Ganesan, aganesan@uci.edu

Corresponding Author

Mihaela Balu

mbalu@uci.edu

Ph: 949-824-8797

Fax: 949-824-8413

Running title:

FLAME: Macroscopic imaging with microscopic resolution

Supplementary Material

Table S1. Summary of features of the FLAME system and existing commercial imaging devices for high-resolution, non-invasive, label-free clinical skin imaging

	RCM	OCT	MPM	
Device (Company)/Features	Vivascope (Caliber I.D., USA; Mavig, Germany)	VivoSight (Michelson Diagnostics, UK)	DermaInspect, MPTflex (JenLab, Germany)	FLAME
Lateral Resolution	< 1.25 μm	<8 μm	< 1 μm	< 1 μm
Axial Resolution	< 5 μm	<5 μm	2 μm	3.3 μm
FOV	500 x 500 $μm^2$	6x6 mm^2	250x250 $μm^2$	1 x 1 mm^2
Maximum scanning area	8x8 mm^2 (mosaic)	6x6 mm^2	250x250 $μm^2$	10x12 mm^2 (mosaic)
Imaging Depth	200-300 μm	1-2 mm	200-300 μm	200-300 μm
Imaging time per unit area	1 s/mm^2	0.01 s/mm^2	96 s/mm^2	2 s/mm^2

Table S2. Imaging parameters of the different modalities of FLAME.

Imaging parameters/ FLAME modality	Scanning area/volume	Lateral Resolution	Imaging time (scanning + CARE processing)
Single field of view (survey)	900x900 $μm^2$	0.9 μm	1-2 s
Single field of view (high resolution)	<500x500 $μm^2$	0.5 μm*	1-2 s
Centimeter scale strip mosaic	12x10 mm^2	1.2 μm	1 min 45 s + 30 s
Sub-centimeter scale tile mosaic	4.5x4.5 mm^2	0.9 μm	1 min 50 s + 50 s
Volumetric image stack (survey)	900x900x150 $μm^3$	0.9 μm	1 min + 1 min
Volumetric image stack (high resolution)	500x500x150 $μm^3$	0.5 μm*	1 min

*Optics-limited resolution

Movie 1: Rapid, millimeter-scale *ex vivo* MPM imaging of human facial skin with sub-micron resolution. Movie showing a lateral browsing of a high resolution tile mosaic with digital zoom highlighting micron-sized melanocytic dendrites that cannot be visualized in the fully zoomed out images. This is a 6.3 x 6.3 mm^2, 49 MPx image acquired and restored with CARE in 3 minutes.

Movie 2: Rapid, sub-micron resolution, *ex vivo* volumetric MPM imaging of scarred facial skin. A z-stack of en face images at different depths covering a volume of 900 x 900 x 150 $μm^3$ sampled every 5 μm from the epidermis to papillary dermis. The stack of 30 1 MPx frames was acquired in 60 seconds. The stack reveals normal distribution of keratinocytes in the epidermis, pigmented keratinocytes (bright green) surrounding the dermal papilla (blue) at the DEJ and probably inflammatory response to scarring (outlined area) in the papillary dermis.

Movie 3: Sub-micron resolution, *ex vivo* volumetric MPM imaging of facial skin tissue. A z-stack of en face images (900 x 900 x 150 $μm^3$, 1 MPx) sampled every 5 μm acquired with 10 seconds per depth (70 frame accumulations) to validate the observed melanocytes with high SNR (~30). Melanocytic dendrites can be visualized at multiple planes in close vicinity of a hair.

This stack was acquired in the sample shown in Fig. 6a at the same location as the inset in Fig. 6e.

Movie 4: Rapid, sub-micron resolution, time-resolved SPC *in vivo* volumetric MPM imaging of human skin forearm. (a) Z-stack of en face images at different depths covering a volume of 540 x 540 x 150 μm^3 sampled every 5 μm from the stratum corneum to papillary dermis. The 1MPx frames within the stack were acquired in 60 seconds. The stack reveals a bright stratum corneum with normal distribution of non-pigmented keratinocytes (green) in the epidermis and pigmented keratinocytes (magenta) surrounding the dermal papilla(blue) at the DEJ. Occasional fibroblasts (green) can be observed in the deeper layers of the papillary dermis. (b) The 3D melanin distribution corresponding to the stack shown in A, obtained by correcting for the red-green channel overlap.

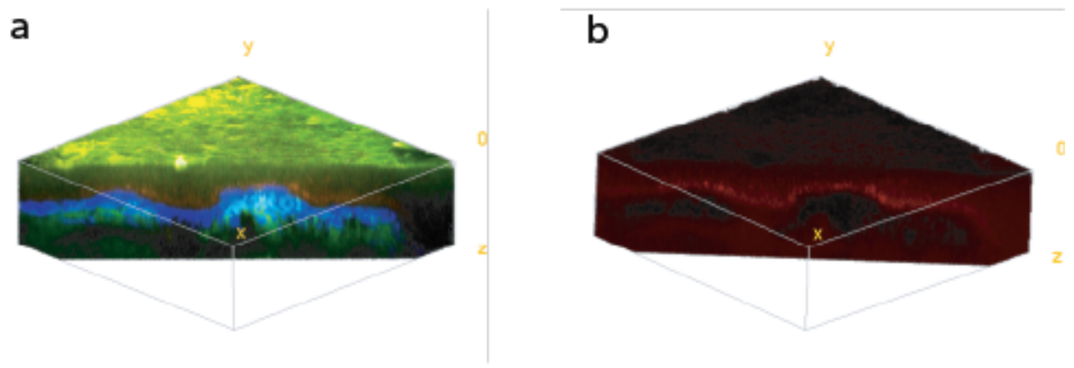

Fig. S1. Rapid, sub-micron resolution, time-resolved SPC *ex vivo* volumetric MPM imaging of human scalp skin tissue. Volumetric representations of a 540 x 540 x 150 μm^3 z-stack of 1 MPx frames, sampled every 5 μm and acquired in 60 seconds. (a) xyz-slice into the volume showing the undulations of the dermal epidermal junction. (b) 3D distribution of melanin obtained by correcting for the channel overlap.

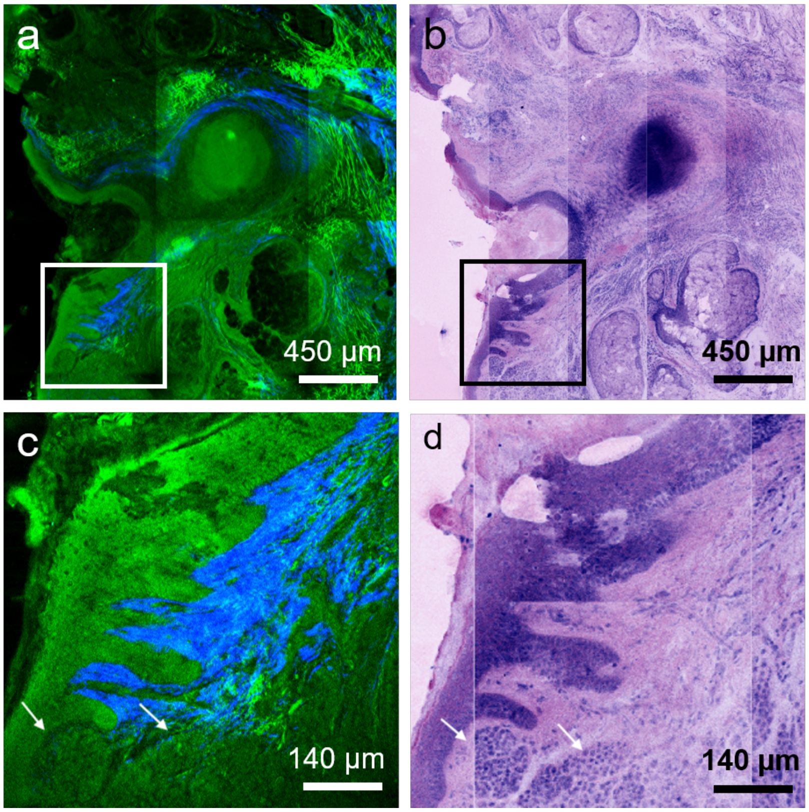

Fig. S2. Thick cross section (xz cut) of a freshly excised human skin tissue (compound nevus). (a,c) MPM images of the unstained tissue. (b,d) Images of the same tissue stained with 40 μg/mL acridine orange (AO) and 40 μg/mL sulforhodamine 101 (SR101) to selectively visualize cell nuclei and cytoplasm respectively. The tissue was imaged with the MPM FLAME system (780 nm excitation, 540/40 and 650/60 nm emission for AO and SR channels) and false-colored into H&E colors (see Ref. 25 for more details). Overall tissue architecture in MPM and H&E-like images can be correlated on large millimeter-scale areas (a,b) and in close-up images (a,b) to better visualize the nests of nevus cells (arrows).

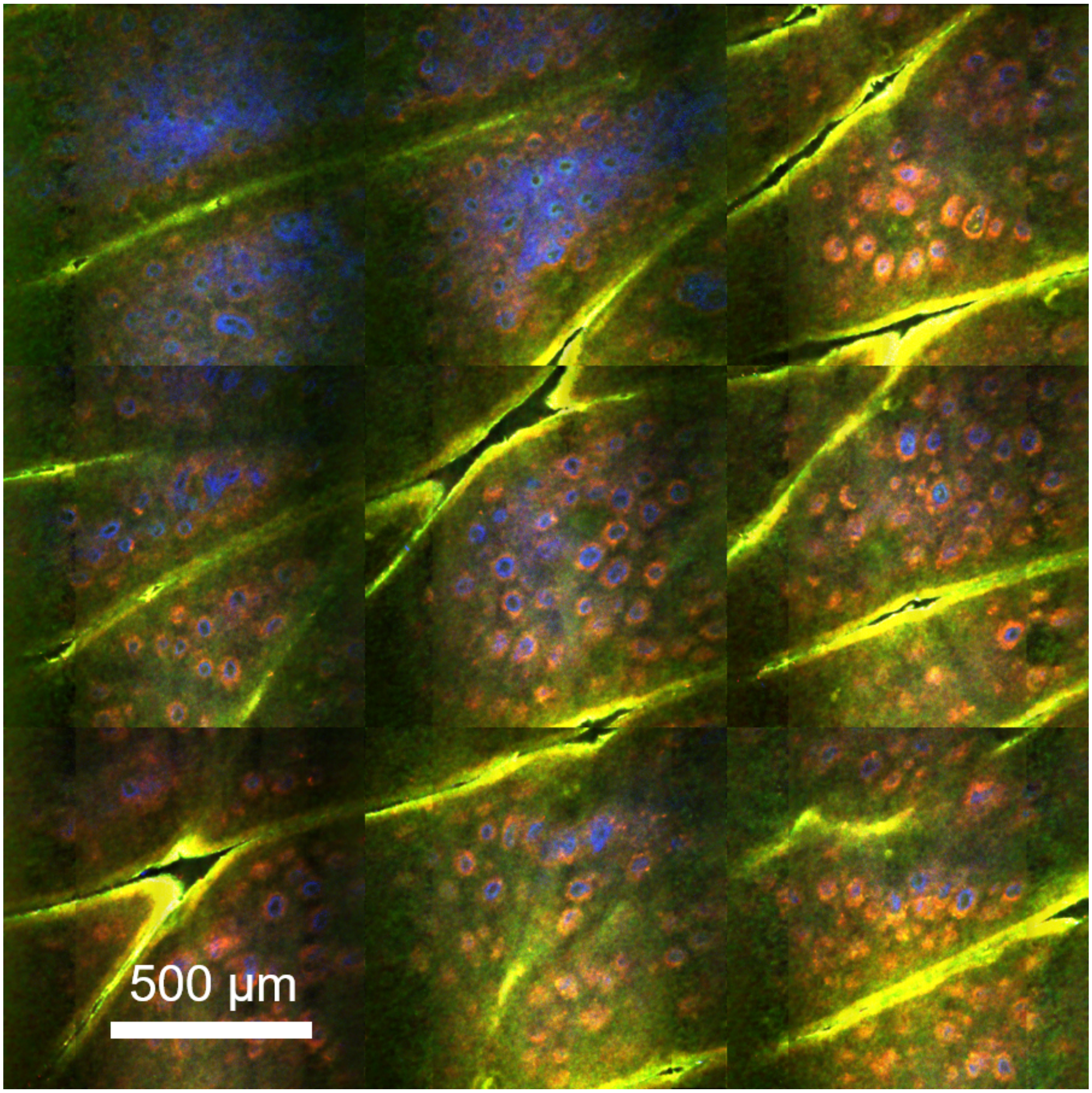

Fig. S3. Rapid, millimeter scale, sub-micron resolution, time-resolved SPC *in vivo* MPM imaging of human skin forearm. Tile mosaic image (2.7 x 2.7 mm^2, 9 MPx) acquired and restored in 45 seconds, at 45 µm below the surface at the DEJ. The image shows a map overview of normal skin morphology at this depth mainly consisting of pigmented keratinocytes (red) surrounding dermal papilla (blue).

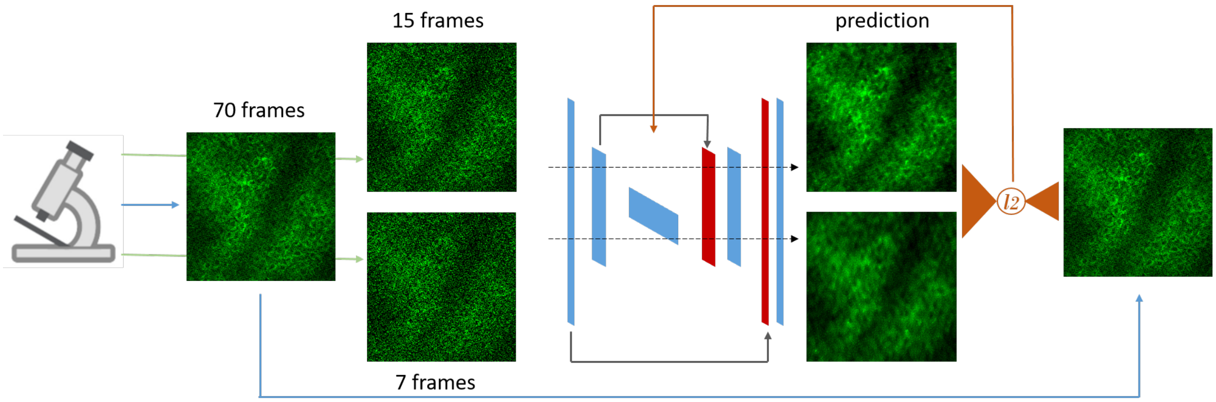

Fig. S4. CARE training flowchart. TPEF images (0.9 x 0.9 mm^2, 1 MPx) are acquired as sequential frames at 8 Hz within the epidermis of freshly excised human skin. Full image sequence consisting of 70 frames (~10 s acquisition time) is accumulated to yield a single image that serves as the ground truth. First 7 and 15 frames of the sequence are summed up and saved to serve as the network input. Images are divided into patches, pooled together and randomly split 90:10 into training and validation sets. High SNR images serve as ground truth and low SNR images as inputs to the CARE neural network. Normalized root mean squared error (NRMSE) is used to determine the model's performance and train the image restoration model with gradient-based optimization[36].

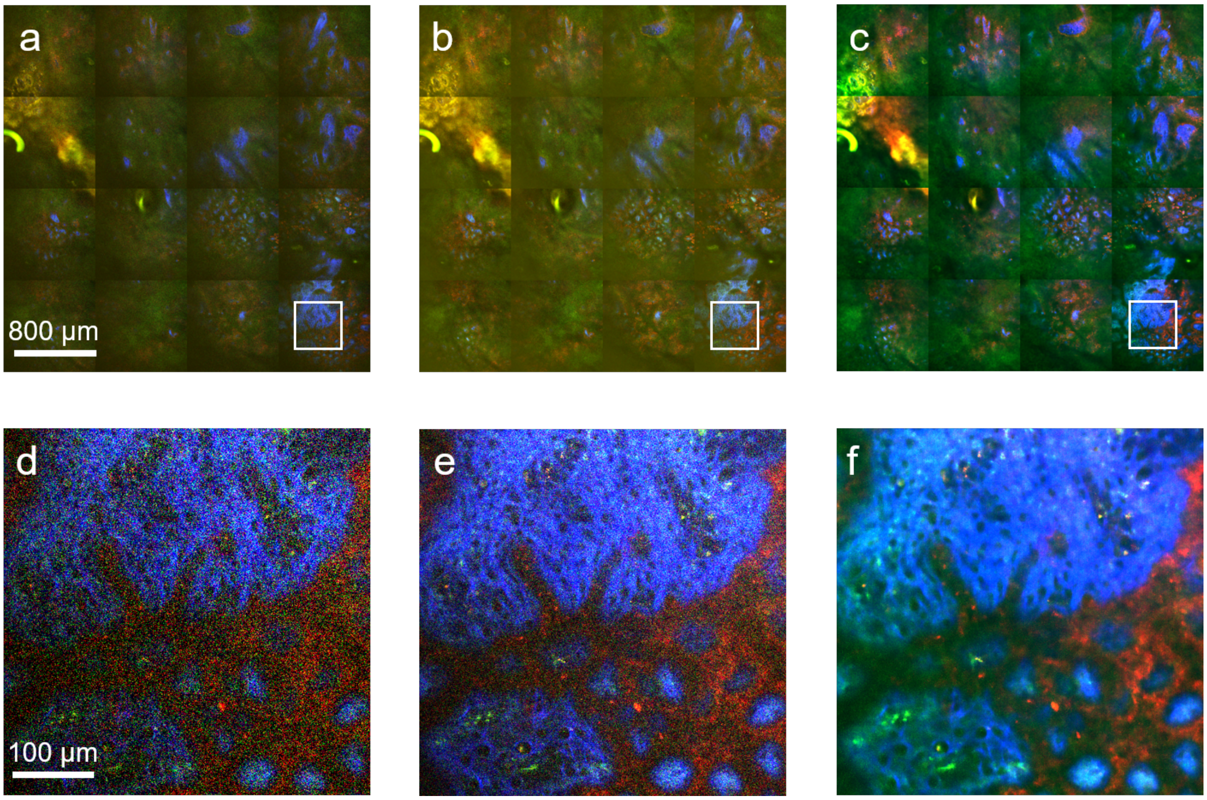

Fig. S5. CARE restoration performance on the independent test set. Tile-mosaic images (3.6x3.6 mm^2, 16 MPx) acquired at the DEJ of a freshly excised human skin sample representing: (a) the network input image (15 accumulated frames), (b) the ground truth image (70 accumulated frames), (c) post-CARE processing image (15 accumulated frames) (d,e,f) 450x450 μm^2 images corresponding to the insets in (a,b,c) show the relative SNR levels demonstrating significant noise reduction and overall similarity with ground truth post neural network processing.

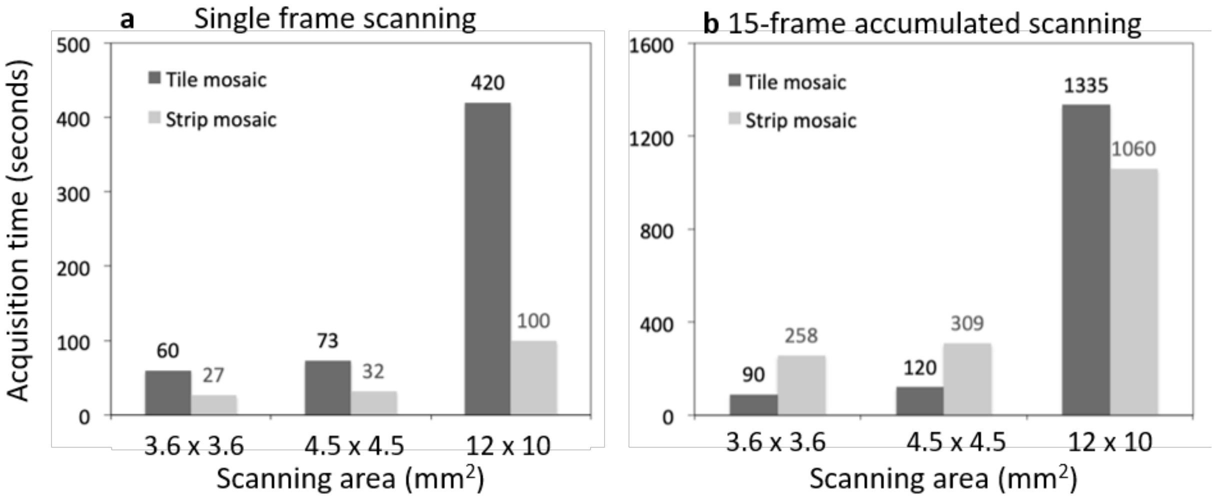

Fig. S6. Acquisition time for different scanning areas with strip (light grey) and tile (dark grey) mosaic schemes. Acquisition times for (a) single frame scanning and (b) 15-frame accumulated scanning corresponding to the scanning areas commonly used in our experiments.
